# Strengthening health promotion at the local level: insights from Swiss municipalities (canton of Vaud) across urban and rural contexts

**DOI:** 10.3389/ijph.2026.1609819

**Published:** 2026-08-10

**Authors:** Maria Lagomarsino, Loreene Casteyde, Emilie Obert, Emmanuelle Garcia, Karin Zürcher, Stéphanie Pin

**Affiliations:** Department of Health Promotion and Prevention, Unisanté, University Center for Primary Care and Public Health, Lausanne, Switzerland

**Keywords:** cluster analysis, health in all policies (HiAP), health promotion, local governance, survey

## Abstract

**Objectives:**

In response to growing interest in local action to promote public health, this study explores how municipalities perceive their role in health promotion (HP), how far HP is integrated into municipal policies, and what support is needed to strengthen local action.

**Methods:**

An online survey was distributed to 300 municipalities in the Canton of Vaud (February - April 2025). Complete responses from 110 municipalities (39% response rate) were included in the analyses. An adaptation of the Maturity Model of Health in All Policies (MM-HiAP) guided the cluster analysis used to identify profiles of municipal engagement in HP.

**Results:**

Four clusters were identified: Not engaged (39%), Relying on others (26%), Engaged but isolated (19%), and Engaged and somewhat structured (16%). Among the key factors influencing HP integration, municipal size and the presence of committed local leadership emerged greatly. Moreover, clusters revealed specific resources and needs, suggesting that support should be adapted to these specific needs.

**Conclusion:**

The study provides a framework for strengthening municipal engagement in HP along a maturity continuum, with implications for policy, practice, and future research across diverse local contexts.

## Introduction

Non-communicable diseases (NCDs) account for over half of premature deaths in Switzerland [1]. Evidence shows that reducing the incidence and burden of NCDs requires not only prevention efforts focusing on behaviour change, but to address social, economic, and environmental determinants of health as well [2–6]. Such comprehensive approaches can improve population health and help curb healthcare costs [7, 8]. Consequently, growing emphasis is placed on interventions that shape wider health determinants–such as education, social inclusion, urban planning, and economic and occupational environments to create settings that support healthy choices and reduce health inequalities [7–10].

Local authorities are widely recognised as pivotal settings for public health and health promotion (HP) due to their capacity to influence both physical environments and community engagement [11, 12]. Furthermore, participation and population empowerment can, in turn, generate significant and lasting impacts on population wellbeing and reducing health disparities [10, 12, 13]. Internationally, this recognition has led to the development of policy frameworks and programmes that explicitly encourage local-level action at the city or municipal level [14–16]. In Switzerland, strategic documents recognise municipalities as key partners in implementing local public health objectives both at national and cantonal level [7, 17]. Access to funding often depends on implementing structural measures emerging from population needs, underscoring the importance of locally tailored approaches [18, 19].

Despite this growing recognition, the role of municipalities in HP is strongly influenced by the characteristics of the Swiss federal system, which lacks a comprehensive national legal framework for public health [20]. As a result, the allocation of health-related responsibilities among the Confederation, the cantons, and the municipalities remains only partially defined and varies considerably across Switzerland.

This variability translates into unequal local capacity to engage in comprehensive HP activities and partly explains differences in the scope and extent of municipal engagement in HP across the country. In many cases, muncipal competences are limited and often exclusively ensuring access to health and social serveces [21–23]. This heterogeneity is further exacerbated by other contextual factors—including, for example, municipal size, urban or rural setting and population characteristics [15, 23, 24]—as well as individual and municipal factors, such as mobilisation of political and administrative actors, along with financial and human resources [21–23, 25].

In summary, although the central role of local authorities in HP is widely acknowledged world-wide and in Switzerland, and key challenges for implementing HP actions at the local level have been identified, there remains a lack of structured and systematic evidence on how HP is actually integrated within municipalities. Moreover, the factors associated with lower or higher levels of HP integration at the municipal level in Switzerland are only partially understood and are often inferred.

Against this backdrop, a more in-depth understanding of how municipalities perceive and integrate HP into their actions and policies appears warranted. Such evidence is needed to identify key levers, barriers, and enabling conditions, as well as municipalities’ needs and expectations in terms of support and capacity building. Our study was designed to address these gaps within the context of the canton of Vaud. The Maturity Model of Health in All Policies (MM-HiAP) by Storm and colleagues [26] served as the conceptual framework guiding the research, that aims to generate practice-oriented knowledge on how to increase HP efforts at the municipal level.

## Methods

The objective of the study is to answer the following research questions: a) how municipalities in the canton of Vaud perceive and integrate HP into their actions and policies; b) what the key levers, barriers, and enabling conditions, as well as municipalities’ needs and expectations are to increase municipalities’ engagement in HP. To answer our research questions, we employed a mixed-methods approach using a sequential explanatory design [27], in which qualitative findings were used to interpret and contextualise the quantitative results. In this article, however, we report exclusively the survey findings (quantitative phase).

### Theoretical framework

To analyse the degree of integration of HP and prevention at the municipal level, the study draws on the MM-HiAP. This model was developed by Storm and colleagues [26] to assess and monitor the integration of the “Health in All Policies” approach within Dutch municipalities and was subsequently adapted to the French context by Porcherie and colleagues [28]. The model distinguishes five levels of maturity, or degrees of integration, of health promotion and prevention at the local level: *recognised, considered, implemented, integrated, and institutionalised.*

**FIGURE 1:**
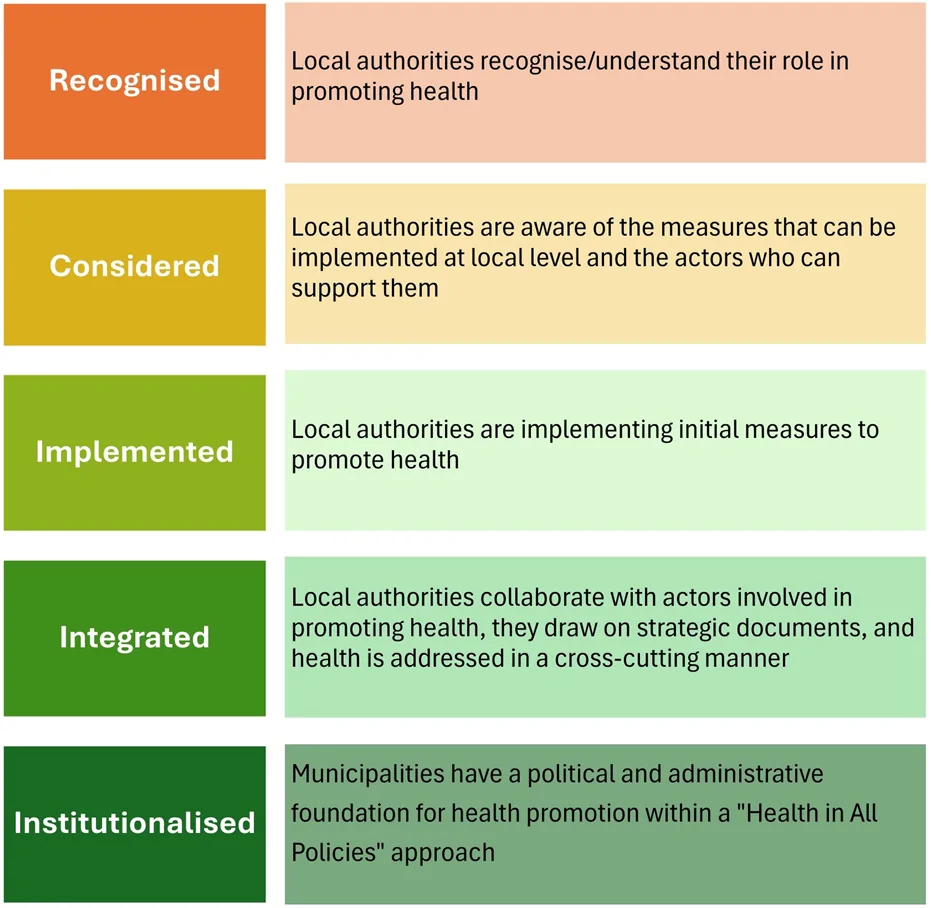
Maturity model for the integration of health promotion at the local level, adapted from the Maturity Model of Health in All Policies (MM-HiAP) from Storm and colleagues [26] (Switzerland, 2026).

Our adaptation to the Swiss context, and more specifically to the canton of Vaud and its municipalities, treats the levels as dimensions (see Figure 1). The aim is therefore to describe the elements corresponding to each dimension based on the information collected from the municipalities, and then to group municipalities in homogenous clusters according to these dimensions.

#### Data collection method

The adapted maturity model was used to design an online questionnaire. The questionnaire was distributed by email from February to April 2025 to all 300 municipalities in the canton of Vaud and it took roughly 40 min to complete. The canton of Vaud is 4th largest Swiss canton (26 cantons in total), occupies 7.8% of national territory, and had 823,881 inhabitants in December 2021 [29]. The questionnaire targeted both political and administrative representatives to maximise participation. Multiple responses per municipality were allowed and later recoded to yield one consolidated response per municipality (see Supplementary Material 1 for the consolidation strategy adopted and Supplementary Table S1.1 for and overview of the respondents’ profile). To maximise participation, no questionnaire items were made mandatory.

#### Measures

At the outset, participants were provided with an overview of the study and informed that answering the questionnaire implied consent to the use of their data in anonymised form. The name of the municipality was collected. Subsequently, the questionnaire was structured into several thematic sections designed to gather data on each component of the maturity model (see Figure 1). Additional items addressed perceived barriers and facilitating factors to engage in HP, as well as municipalities’ needs in this area. The questionnaire concluded with socio-demographic questions. The questionnaire is available in French and English (translated) in the institutional data repository (https://doi.org/10.16909/dataset/65).

Based on the operationalisation the model’s dimensions (see Supplementary Material 1, Supplementary Table S1.2), respondents were asked to indicate the level of responsibility attributed to various actors for implementing HP measures, as well as the relative importance their municipality attaches to HP compared with other policy priorities (*Recognised dimension*). Participants reported whether they were aware of the support services available to municipalities in relation to HP, and whether they were familiar with federal or cantonal strategies and plans relevant to HP (*Considered dimension*). Respondents indicated which types of measures had been implemented in their municipality (*Implemented dimension*), covering three categories: information and awareness raising activities; interventions targeting the built and natural environment; and a broad set of interventions for specific population groups aimed at promoting healthy lifestyles, social interaction, and social cohesion.

For the *Integrated dimension*, respondents were asked whether they actually use–rather than merely know–the elements of the *Considered dimension* (support services and documentations). They also reported the extent to which impacts on public health are taken into account when implementing measures across different thematic areas, such as buildings, mobility and transport, climate and energy.

Finally, the questions related to the *Institutionalised dimension* included whether the municipality has municipal plans or policies in relevant thematic areas, whether staff members are specifically dedicated to these topics, and whether networking measures involving actors or partner organisations are implemented in the municipality. In the Supplementary Material 1, we report the translated version of the questions and describe dimension-specific thresholds to determine whether a municipality meets a given dimension.

### Data analysis

All analyses were conducted using R version 4.4.2 (2024-10-31 ucrt) within a Quarto project environment, and were performed in three steps: (1) calculation of the score for each dimension of the maturity model, resulting in 5 dichotomous variables (detailed information is available in Supplementary Material 1), (2) identification and clustering of municipal segments, and (3) description of the clusters by dimensions of the maturity model and comparison of their barriers, facilitators, and needs. The database and the code are available in the institutional data repository (https://doi.org/10.16909/dataset/65).

#### Clustering

To assess methodological robustness, hierarchical agglomerative clustering was performed using three combinations of distance measures and linkage methods [30–32]: Hamming distance with average linkage, Gower distance with average linkage, and Gower distance with Ward’s linkage method. The analysis and visual inspection of the corresponding three dendrograms consistently suggested a four-cluster solution. The final solution based on Gower distance and Ward’s linkage was selected on the basis of the highest mean silhouette width (mean silhouette width of 0.54 for the 4 clusters solution retained) and superior interpretability of the resulting cluster profiles. Although Ward’s linkage is suited for Euclidean distances, simulation studies on clinical data have supported its use with Gower distance in hierarchical clustering, reporting superior cluster recovery across data types [33].

#### Cluster description and comparisons

We then examined how the clusters could be meaningfully described by the maturity model and municipalities’ characteristics (size, type and region). We ran MANOVAs [34], ordinal logistic regression and non-parametric tests [35] to comparing distributions of key variables across clusters (see Supplementary Material 2 for the match of variables and the statistic test performed).

## Results

### Participants

We collected 250 responses to the questionnaire, representing 170 municipalities. Among them, 140 people, representing 119 municipalities, completed the full questionnaire. 110 municipalities have answered to all questions regarding the variables for cluster analysis as no question was mandatory (see Data collection method section and Supplementary Material 1). The total sample was representative with regard to municipality’s region, and type (see Table 1). Municipalities with fewer than 1,000 residents appear to be slightly underrepresented (44% versus 53%).

**TABLE 1:** Comparison of the questionnaire sample and canton of Vaud (Switzerland, 2026).

​	Questionnaire sample	Sample used for clustering	Canton de Vaud
	N = 119	N = 110	N = 300
Characteristic	% (n)	% (n)	% (n)
Size			
Less than 1′000 inhabitants	44% (52)	45% (50)	53% (158)
1′000–2′999	25% (30)	25% (28)	27% (81)
3′000–9′999	22% (26)	21% (23)	15% (44)
10′000 or more	9% (11)	8% (9)	6% (17)
Type			
Rural	50% (60)	53% (58)	54% (163)
Intermediate	24% (28)	23% (25)	27% (80)
Urban	26% (31)	25% (27)	19% (57)
Region			
South	8% (9)	7% (8)	9% (27)
West	25% (30)	25% (25)	29% (87)
Nord	45% (54)	48% (53)	42% (126)
East	22% (26)	22% (24)	20% (60)

### Description of clusters

Table 2 presents descriptive statistics for the full sample as well as the profiles of the retained four clusters, showing the proportion of municipalities that complied with each of the five maturity dimensions (see Figure 1). Overall, most of the 110 municipalities reached the *Recognised dimension* (78%), with progressively fewer reaching higher maturity levels, reflecting limited full integration and institutionalisation of HP.

**TABLE 2:** Proportion of municipalities that complied to each of the five dimensions of the maturity level within the four clusters and the sample (Switzerland, 2026).

Dimension	Cluster 1: “Not engaged” N = 43	Cluster 2: “Relying on others” N = 28	Cluster 3: “Engaged but isolated” N = 21	Cluster 4: “Engaged and somewhat structured” N = 18	Sample used for clustering N = 110
	% (n)	% (n)	% (n)	% (n)	% (n)
Recognised**	58% (25)	89% (25)	90% (19)	94% (17)	78% (86)
Considered***	-	79% (22)	-	100% (18)	36% (40)
Implemented***	-	-	100% (21)	100% (18)	35% (39)
Integrated***	-	46% (13)	33% (7)	72% (13)	30% (33)
Institutionalised***	-	-	-	28% (5)	5% (5)

* p-level <0.10. ** p-level <0.05. ***p-level <0.001.

The four-cluster solution chosen highlights distinct profiles (see Table 2). The largest cluster, Not engaged (Cluster 1, N = 43), included municipalities complying exclusively to the *Recognised dimension*–i.e., 58% of the municipalities complied to the *Recognised dimension* and none to the other dimensions. At the other extreme, the smallest cluster, Engaged and somewhat structured (Cluster 4, N = 18), comprised the most mature municipalities, which not only recognised their role but also actively promoted health–both independently and in partnership. A few among them institutionalised these initiatives through a health plan and dedicated public health staff (28%). Relying on others (Cluster 2, N = 28) included municipalities that addressed these topics mainly by delegating responsibilities to external actors. Finally, Engaged but isolated (Cluster 3, N = 21) grouped municipalities that recognised their role in promoting health but acted largely alone using their own resources.

To formally assess these differences, chi-square and Fisher’s exact tests were conducted for each of the five dichotomous dimensions, with cluster membership serving as the grouping variable. The global tests indicated that cluster membership was significantly associated with all dimensions: *Recognised* (Fisher’s exact test, *p* = .001; Cramer’s V = 0.39), *Considered* (χ^2^ (3) = 89.63, *p* < .001; Cramer’s V = 0.90), *Implemented* (χ^2^ (3) = 110.00, *p* < .001; Cramer’s V = 1.00), *Integrated* (χ^2^ (3) = 37.42, *p* < .001; Cramer’s V = 0.58), and *Institutionalised* (Fisher’s exact test, *p* < .001; Cramer’s V = 0.49). These results confirm that the clusters differ significantly on each dimension overall, with effect sizes ranging from moderate to very large, indicating substantial differences across clusters. Further analyses aimed at identifying which specific pairwise cluster differences were statistically significant proved challenging due to the presence of numerous zero cells across several dimensions. The presence of zero cells in several clusters indicates near-complete separation, which inflates global test statistics but simultaneously prevents stable estimation of pairwise contrasts. That is why we described these differences in a descriptive manner above.

As regards the *Implemented dimension*, and irrespective of whether the municipalities in the different clusters comply with it, it is interesting to examine whether municipalities are implementing HP activities and, if so, which ones. Notably, 109 out of 110 municipalities have implemented at least one activity out of the 19 types of activities listed in the questionnaire, but the number of measures implemented varies considerably from one municipality to another (M = 8, SD = 4.71), see Supplementary Material 3 for more information. A multivariate analysis of variance (MANOVA) revealed significant differences in term of implemented activities across clusters (Wilks’ Λ = 0.27, F(9, 253.26) = 19.97, p < .001). Post-hoc comparisons indicated a clear gradient across clusters, with the Engaged and somewhat structured (M = 14, SD = 3.20) and Engaged but isolated (M = 11, SD = 3.16) clusters generally exhibiting significantly higher number of implemented measures of the three types than the Not engaged (M = 8, SD = 3.91) and Relying on others (M = 5, SD = 2.99) clusters.

Concerning municipalities’ characteristics (see Table 3), the distribution of municipality size varied across the clusters (ordinal logistic regression, χ^2^ (3) = 23.44, p < .001). Post-hoc comparisons revealed that municipalities in the Not engaged cluster were significantly more likely to be small municipalities of less than 1′000 inhabitants compared with Engaged but isolated (p = .014) and Engaged and somewhat structured clusters (p < .001), and those in Relying on others cluster were significantly more likely to be small municipalities of less than 1′000 inhabitants than those in Engaged and somewhat structured cluster (p = .017). Other pairwise differences were not statistically significant after Bonferroni correction.

**TABLE 3:** Descriptive statistics of municipalities’ characteristics within the four clusters (Switzerland, 2026).

Characteristic	Cluster 1: “Not engaged” N = 43	Cluster 2: “Relying on others” N = 28	Cluster 3: “Engaged but isolated” N = 21	Cluster 4: “Engaged and somewhat structured” N = 18
​	% (n)	% (n)	% (n)	% (n)
Size ***				
Less than 1′000 inhabitants	63% (27)	50% (14)	29% (6)	17% (3)
1′000–2′999	28% (12)	21% (6)	33% (7)	17% (3)
3′000–9′999	9% (4)	25% (7)	19% (4)	44% (8)
10′000 or more	0% (0)	4% (1)	19% (4)	22% (4)
Type ***				
Rural	65% (28)	64% (18)	48% (10)	11% (2)
Intermediate	26% (11)	21% (6)	10% (2)	33% (6)
Urban	9% (4)	14% (4)	43% (9)	56% (10)
Region				
South	5% (2)	4% (1)	10% (2)	17% (3)
West	23% (10)	25% (7)	29% (6)	11% (2)
Nord	56% (24)	54% (15)	33% (7)	39% (7)
East	16% (7)	18% (5)	29% (6)	33% (6)

* p-level <0.10. ** p-level <0.05. ***p-level <0.001.

Moreover, the distribution of municipality’s types differs significantly across clusters, as indicated by a chi-square test with Monte Carlo simulation (χ^2^ = 25.85, *p* < .001; Cramer’s V = 0.34). The analysis of standardised residuals revealed that the Not engaged cluster was characterised by a significant over-representation of rural municipalities and an under-representation of urban ones, while the Engaged and somewhat structured cluster exhibited the opposite pattern, with substantially more urban and fewer rural observations. The Engaged but isolated cluster also showed a significant over-representation of urban municipalities, although to a lesser degree, whereas the Relying on others cluster did not deviate strongly from expected frequencies (see Supplementary Table S2.1 in the Supplementary Material 2 for detailed results). In contrast, the distribution of region categories did not differ significantly across the clusters (χ^2^ = 9.03, *p* = .448; Cramer’s V = 0.17), indicating that municipalities’ region did not vary systematically by cluster.

### Barriers/facilitators and needs

#### Barriers/facilitators

**FIGURE 2:**
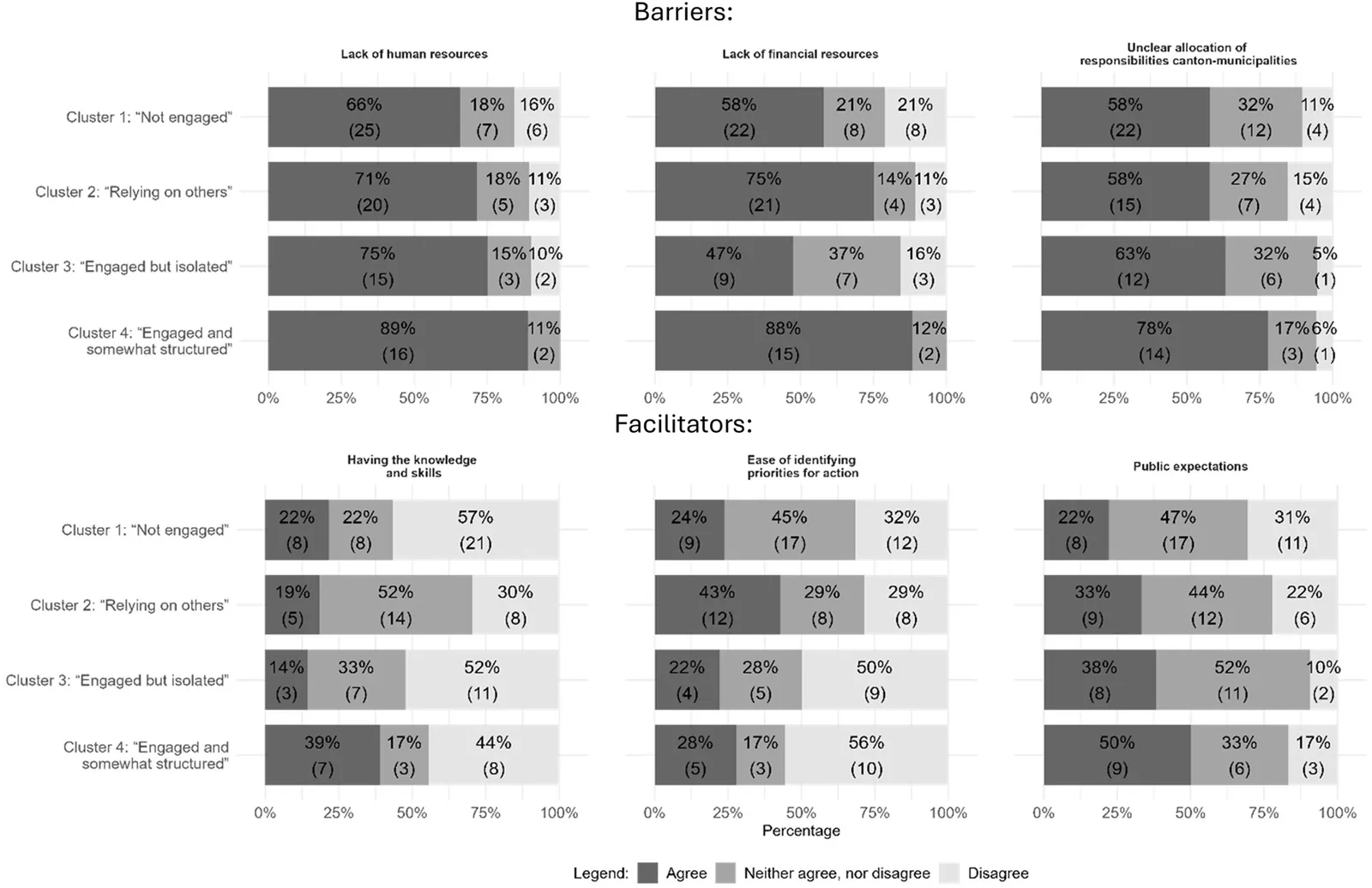
Self-report agreement to identified barriers and facilitators for municipal engagement in health promotion (Switzerland, 2026).

Across the four clusters, respondents expressed consistently high levels of agreement regarding the main barriers examined in the survey (see Figure 2). Perceptions of a lack of human resources were particularly widespread: agreement ranged from 66% in the Not engaged cluster to 89% in the Engaged and somewhat structured cluster. A similar pattern emerged for financial constraints, with one notable exception: in the Engaged but isolated cluster, fewer than half of the municipalities only agreed that financial resources were insufficient. Perceptions of unclear allocation of responsibilities between the canton and the municipalities were also prevalent, with agreement levels ranging from 58% to 78% across clusters.

In contrast, responses to the facilitator items revealed more nuanced differences across clusters (see Figure 2). Municipalities in the Engaged and somewhat structured cluster mostly reported having the necessary knowledge and skills, however they often reported difficulties in identifying priorities for action. The results further suggested that municipalities in this cluster were motivated by public expectations (50% agreement). Conversely, municipalities in the Not engaged cluster showed low levels of agreement across all three facilitators, most notably with respect to lacking the necessary knowledge and skills (57% disagreement). Moreover, those in the Relying on others cluster reported more often ease in identifying priorities for action (43% agreement) and many neither agreed nor disagreed with having the required skills (52%), likely reflecting their reliance on external actors. Municipalities in the Engaged but isolated cluster reported difficulties in identifying priorities for action (50% disagreement) and indicated a lack of knowledge and skills (52% disagreement), despite otherwise demonstrating engagement.

#### Needs

**FIGURE 3:**
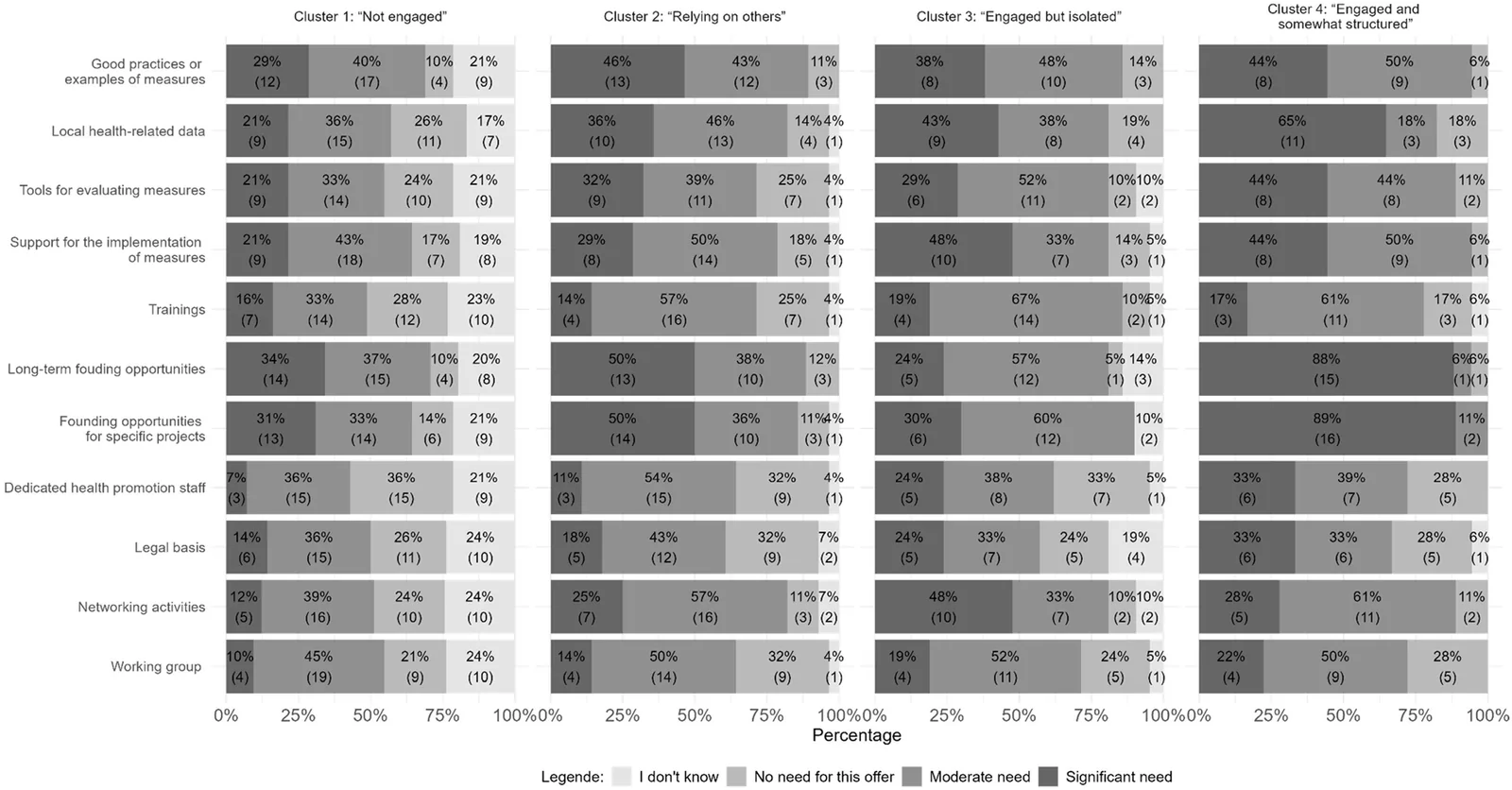
Self-report assessment to identified needs for municipal engagement in health promotion (Switzerland, 2026).

Across clusters, municipalities expressed systematically different levels of need for support and resources related to HP (see Figure 3). The Not engaged cluster consistently reported the lowest levels of need and the highest shares of respondents indicating uncertainty (“I don’t know”). On the opposite, the Engaged and somewhat structured cluster showed the most pronounced needs.

For the municipalities belonging to the Not engaged cluster, the most prominent needs were long-term fundings (34%), followed by fundings for specific projects (31%), and access to good practices or examples of interventions (29%). The Relying on others cluster showed its strongest needs in financial assistance for specific projects (50%) and long-term financial assistance (50%), with good practices next (46%). In the Engaged but isolated cluster, the top needs were support for implementation (48%) and networking activities (48%), followed by access to data on health or social cohesion (43%). Finally, the municipalities belonging to the Engaged and somewhat structured cluster needed mostly financial assistance for specific projects (89%), long-term financial assistance (88%), and access to data on health or social cohesion (65%).

## Discussion

This study provides new evidence on how municipalities perceive, organize, and allocate resources for local HP, and how these features cluster along a maturity continuum. Using an adaptation of the MM-HiAP [26, 28], we identified four distinct municipal profiles in the canton of Vaud–Not engaged, Relying on others, Engaged but isolated, and Engaged and somewhat structured–that differ markedly across the five maturity dimensions *(Recognised, Considered, Implemented, Integrated, Institutionalised)*, the volume and mix of implemented activities, and the pattern of expressed needs.

The four profiles map coherently onto the original MM-HiAP work [26, 28]: progression in maturity is accompanied by shifts from mere recognition for the majority of municipalities (Not engaged) and ad-hoc initiatives (Relying on others and Engaged but isolated) to structured governance (e.g., plans, dedicated staff) and routine health considerations in non-health policies (Engaged and somewhat structured). According to the litterature, municipality’s engagement in HP is closely linked to its size and ressources [25, 36]. Not surprisingly, the most mature cluster in our sample–the smallest–groups the biggest, mostly urban and most resourceful municipalities. This cluster self-reports the needs for mostly long-term financing and data to prioritize initiatives, consistent with institutions that have the capacity to act and now seek to consolidate and scale efforts through sustained resourcing and evidence support. Conversely, municipalities in the Not engaged cluster, which are the smallest and predominantly rural municipalities, report lower levels of significant need overall and higher uncertainty (“I don’t know”), suggesting that foundational awareness, guidance, and sharing of good practices remain the priorities at this end of the continuum.

Particularly noteworthy are the clusters Relying on others and Engaged but isolated, which group together municipalities of varying sizes, type (rural/urban), and resource levels that have nevertheless adopted markedly different–almost opposite–approaches. The former primarily outsource their activities, relying on services provided by local partners, whereas the latter operate within their means (lowest needs of financial support), underscoring the challenges of accessing external support. The literature identifies several factors that may help explain these differences. For the first, regions characterised by a high density of relevant local actors may offer a more favourable environment for the development and expansion of HP measures, fostering a virtuous cycle in which existing activities stimulate further initiatives and collaborations [25, 37]. For the second, rich municipalities more easily invest in HP; and political support or the presence of a particularly committed municipal actors (leaders), can facilitate the advancement of HP initiatives even within limited resourceful municipalities [36, 38, 39].

These differences are also reflected in their expressed needs. Municipalities in the Relying on others cluster combine a high demand for project and long-term financing with a strong interest in good practices, a pattern consistent with externally oriented implementation strategies. In contrast, municipalities in the Engaged but isolated cluster report substantial needs for implementation support, networking, and data, signalling limited connectivity and expert knowledge despite strong internal commitment.

Additionally, although local collaborations and spillover effects between neighbouring municipalities can be observed, these dynamics remain confined to geographically proximate contexts and do not translate into strong regional pattern; accordingly, no substantial differences emerge across regions in terms of cluster composition. These finding supports previous work on policy diffusion, suggesting that proximity facilitates interactions and exchange but does not automatically lead to wider regional convergence [40].

Taken together, our findings suggest that a one-size-fits-all approach to support HP efforts at the local level–that is, in municipalities–is unlikely to be effective, particularly in settings where higher-level leadership is limited. This appears to be the case in Switzerland, which is characterised by a decentralised system and fragmented governance [20]. While national and cantonal frameworks provide guidance and support, they do not assign formal responsibilities to municipalities; consequently, their uptake and translation into local action remain uneven.

First, a stronger strategic framework clarifying municipalities’ responsibilities for HP, together with the resources available to fulfil them, could help strengthen local engagement [41]. Second, differentiated support strategies aligned with municipalities’ level of maturity are needed to reinforce local capacities and facilitate access to support mechanisms adapted to their specific contexts. Because municipal engagement also depends on organisational and local factors, it is important to strengthen the broader support environment surrounding municipalities, including associations, local actors, and partner organisations.

Specifically, to address the full gradient of maturity levels, support could be tailored as follows: (i) Activation packages for Not engaged municipalities (orientation activities and quick-start tools); (ii) Co-implementation support and targeted grants for the Relying on others group; (iii) Networking platforms and experts support for those Engaged but isolated; and (iv) Multi-year programmatic funding, shared data about localised indicators (e.g., health, social cohesion, equity) for the Engaged and somewhat structured municipalities. These support strategies are consistent with international HiAP practices for scaling HP efforts and ensuring their durability [26, 28, 42, 43].

### Limitations

The theoretical framework provided a systematic approach for examining the level of integration of HP at the municipal level. While certain adjustements were introduced to better capture the specific features of the region under study, the framework may have limited the faithful representations of the complexity of the existing system in the canton of Vaud. A complementary qualitative component, currently under analysis by the research team, is expected to offer a more nuanced understanding of contextual factors and barriers that may not have been revealed through the preceding quantitative analyses.

The study aimed not only to assess respondents’ perspectives on HP, but also to develop a more comprehensive understanding of the organisation of the actors system (e.g., canton, municipality, HP specialists)–especially at the municipal level (i.e., elected or staff members)–and to identify structural differences within it. However, the breadth of this approach required certain trade-offs, which limited the depth of analysis that could be devoted to each dimension.

The municipalities that participated in the survey constitute a sample that is representative of Vaud municipalities in terms of size, typology, and region. Considerable efforts were undertaken throughout the data collection process to reach the widest possible diversity of municipalities. Nonetheless, it is likely that participating municipalities were those with greater interest, familiarity, and prior experience in HP. As a result, the findings should be interpreted cautiously.

From a methodological perspective, a few limitations should be acknowledged. Although a structured procedure was used to consolidate multiple responses per municipality, reliance on a single reference respondent for subjective variables may have introduced selection bias, as individual perspectives may not fully represent the municipality as a whole. For example, differences in perceptions and needs may vary according to the respondent’s role within the municipality; however, the available data were insufficient to conduct robust cross-analyses. In addition, because questionnaire items were not mandatory in order to maximise participation, some responses contained missing data. Consequently, only municipalities with complete information could be included in the statistical analyses, slightly reducing the representativeness of the final sample.

### Conclusions

This article advances previous work [11, 12] by testing a method to assess the level of HP integration at the municipal level while simultaneously identifying the specific challenges and needs municipalities face along a maturity continuum. In doing so, it provides actionable insights for stakeholders–such as cantonal authorities and partner organisations–seeking to strengthen local HP efforts through targeted support.

Unlike much of the existing literature, which focuses on large urban settings, our findings are particularly relevant for contexts characterised by small municipalities and the absence of strong centralised regulation, as is the case in Switzerland. In such settings, variability in engagement becomes more visible and informative. Municipal engagement is heterogeneous and often depends on local initiative, resources, and leadership. The barriers as well are context sensitive. Overall, and beyond its methodological contribution, this study broadens the evidence base and emphasises the need for tailored, context-sensitive strategies to support HP in similar governance and territorial configurations.
