# Supplementary material for: Strengthening health promotion at the local level: insights from Swiss municipalities (canton of Vaud) across urban and rural contexts
Source: Int J Public Health. 2026 Aug 10;71:1609819. doi: 10.3389/ijph.2026.1609819 (PMC13501076; doi:10.3389/ijph.2026.1609819)
Supplement: Supplementary file 1 [file Supplementaryfile1.docx]

# Supplementary material 1

**Consolidation strategy for multiple answers:**

In cases of multiple responses from a single municipality, the response from the participant who progressed the furthest in the questionnaire (e.g. completed questionnaires) was selected as the reference. In the event of a tie, a priority criterion based on respondent role was applied (Mayor, Municipal council member, Municipal secretary, then Municipal employee/Other). For subjective variables (e.g. opinions or perceived importance), only the reference response was retained. However, factual information provided by other respondents from the same municipality and missing for in the answer of reference—such as the implementation of measures or awareness of partners—was also incorporated.

**Table SM1.1. Profile of respondents (Switzerland, 2026).**

|  | **Sample Data**  **N = 250***^1^* |
| --- | --- |
| Role |  |
| **Elected officials:** Mayor | 8% (19) |
| **Elected officials:** Municipal council member | 52% (130) |
| **Administrative staff:** Municipal secretary | 14% (36) |
| **Administrative staff:** Municipal employee | 19% (47) |
| **Administration staff:** Other | 7% (17) |
| For how long? |  |
| Less than a year | 10% (26) |
| 1 to 5 years | 50% (125) |
| 6 to 10 years | 21% (52) |
| 11 to 15 years | 9% (23) |
| Over 15 | 9% (22) |
| *^1^ Since incomplete responses are included, the number of responses for each question varies slightly; N represents the maximum number of responses obtained for the category at the sample level* | |

In the questionnaire, a targeted set of items was used to define each dimension of the model (see Table SM1.1) and we specified dimension-specific thresholds to determine whether a municipality meets a given dimension.

| Table SM1.2. List of questions and items (Switzerland, 2026). | | |
| --- | --- | --- |
| Dimension: | **Questions** | **Items** |
| Recognised | - In your opinion, what level of responsibility do the following stakeholders have in implementing measures to promote health and social cohesion at the municipal level? - In your opinion, how much importance does your municipality place on health promotion compared to other issues? | 5-point Likert scale (from *No responsibility to* ***Primary responsibility***)*  14 actors - Municipality was used in the analyses  3-point Likert scale (Not important. Somewhat important, Very important)* |
| Considered | - Are you familiar with the following support services available to municipalities for health promotion? - Are you familiar with the following federal or cantonal strategies and plans? | Options: No, I've never heard of this type of offer; I’m familiar with this type of offer, but I don’t know if the municipality uses it; I am familiar with this type of service, and I know that the municipality does not use it; I am familiar with this type of service and I know that the municipality uses it  18 services - Known to the municipality  Options: No; Yes, I’ve heard of them; Yes, and the municipality has used this document as a basis for its measures or policies  10 documents (strategies or plans)- Known to the municipality |
| Implemented | - Which of the proposed measures have been implemented in your municipality? | Options: Yes; No*  19 types of activities: Information and awareness-raising (3); Interventions in the built and natural environment (4); Interventions for population groups aimed at promoting healthy lifestyles, social interaction and strengthening social cohesion (12), see Supplementary material 3 for more details |
| Integrated | - Are you familiar with the following support services available to municipalities in relation to HP? - Are you familiar with the following federal or cantonal strategies and plans? - In your municipality, are the impacts on public health taken into account when implementing measures on the following topics? | Options: No, I've never heard of this type of offer; I’m familiar with this type of offer, but I don’t know if the municipality uses it; I am familiar with this type of service, and I know that the municipality does not use it; **I am familiar with this type of service and I know that the municipality uses it**  18 services - Used by the municipality  Options: No; Yes, I’ve heard of them; **Yes, and the municipality has used this document as a basis for its measures or policies**  10 documents (strategies or plans)- Used by the municipality  Options: Yes, No*  7 topics: Construction of new buildings, infrastructure and public spaces; Mobility and transport; Climate and energy; Ageing / older people; Family / children / young people; Social services; Integration of foreign nationals and refugees |
| Institutionalised | - Does your municipality have a municipal or inter-municipal plan or policy on the following topics? - Does your municipality employ someone specifically dedicated to the following topics? - Which of the proposed measures have been implemented in your local authority? | Options: Not, Not but in the past, Not but we are thinking about having one, Yes*  6 topics - Health/ public health was used in the analyses  Options: Yes, No*  7 topics - Health/ public health was used in the analyses  Options: Yes; No*  2 types of measures: Establishment by the municipality of cross-departmental working groups on health, social, and public space issues; Workshop/conference/platform/meeting of professionals or community organization representatives |

* The “I don’t know” option was given.

Thresholds (adapted from Porcherie et al., 2022):

- Recognized
  Assigned when the municipality reports “High responsibility” or “Primary responsibility” on the relevant topics and rates the importance given to health promotion as “Very important” or “Moderately important” compared with other local priorities.
- Considered
  Assigned when both (a) the number of support offers known and (b) the number of federal/cantonal strategies or plans known are greater than or equal to the corresponding medians, namely ≥ 11 support offers and ≥ 6 strategies/plans known.
- Implemented
  Assigned when the municipality reports more than one measure in each of the three measure types:
  1. Information and awareness-raising,
  2. Interventions on the built and natural environment,
  3. Interventions targeting population groups to foster healthy lifestyles, social encounters, and strengthen social cohesion.
- Integrated
  Assigned when the municipality reaches or exceeds the medians for:
  (a) support offers used, (b) federal/cantonal strategies or plans used, and (c) municipal actions in which health is explicitly considered, namely ≥ 3 support offers used, ≥ 1 strategy/plan used, and ≥ 5 municipal actions.
- Institutionalized
  Assigned when the municipality (a) has a health or public health plan (ongoing or approved), (b) employs a dedicated public health staff member, and (c) has implemented at least one networking measure (e.g., an interdepartmental working group on health, social issues, or public spaces).

# Supplementary material 2

| Variables | Statistic test |
| --- | --- |
| Dimensions (dichotomous) | Pearson’s chi-squared test or Fisher’s tests (descriptive) |
| Size (ordinal) | Ordinal logistic regression (predictive) |
| Type (categorical) | Pearson’s chi-squared test or Fisher’s test (descriptive) |
| Region (categorical) | Pearson’s chi-squared test or Fisher’s test (descriptive) |
| Detail dimensions: measures (continuous) | Multivariate analysis of variance (MANOVA) |
| Barriers (categorical) | - |
| Needs (categorical) | - |

Standardized residuals help identify which specific typology–cluster combinations contribute to the overall chi‑square significance. Values above |2| indicate cells with significantly more or fewer observations than expected.

**Table SM2.1 Standardized residuals for type by clusters analysis (Switzerland, 2026).**

|  | **Variable : Type** |
| --- | --- |
| **Clusters** |  |
|  |  |

# Supplementary material 3

**Information and awareness-raising (3):**

- Written communication on issues related to health promotion and social cohesion
- One-off events or activities for the population on issues related to health promotion and social cohesion
- Awareness-raising among municipal staff on issues related to health promotion and social cohesion

**Interventions in the built and natural environment (4):**

- Design of public spaces that promote physical activity and social interaction
- Creation of meeting and gathering spaces
- Infrastructure promoting active mobility, traffic calming, and traffic safety
- Creation of housing or neighborhoods that foster social cohesion and well-being

**Interventions promoting healthy lifestyles and strengthening social cohesion (12):**

- Community-based social work (outreach social work)
- Socio-cultural activities and facilitation
- Individual financial assistance
- Financial or logistical support to local associations or resident-led initiatives
- Establishment of seniors’ groups
- Establishment of other population groups to foster social connections
- Socio-professional integration activities
- Health or social drop-in services
- One-off courses to introduce or raise awareness of a specific topic
- Sports or cultural camps for school-aged children
- Weekly physical or creative activity classes
- Development of community-based approaches (assessments and action plans)

**Distribution of activities implemented by municipalities**

| **Table SM3.1. Descriptive statistics of municipalities’ actions within the four clusters (Switzerland, 2026).** | | | | | |
| --- | --- | --- | --- | --- | --- |
|  | **Cluster 1:**  **“Not engaged”**  **N = 43** | **Cluster 2: “Relying on others” N = 28** | **Cluster 3: “Engaged but isolated” N = 21** | **Cluster 4: “Engaged and somewhat structured” N = 18** | **Sample used for clustering**  **N= 110** |
| **Type of activity (n)** | **M (SD)** | **M (SD)** | **M (SD)** | **M (SD)** | **M (SD)** |
| **Information and awareness raising activities (3)** | 1 ( .69) | 1 ( .57) | 2 ( .36) | 2 (0.51) | 1 ( .97) |
| **Interventions for the built and natural environment (4)** | 1 (1.04) | 2 (1.39) | 3 ( .60) | 3 ( .76) | 2 (1.22) |
| **Interventions promoting healthy lifestyles and social cohesion (12)** | 3 (2.31) | 5 (3.07) | 6 (2.78) | 9 (2.43) | 5 (3.27) |
| **N° of activities TOT** | 5 (2.99) | 8 (3.91) | 11 (3.16) | 14 (3.20) | 8 (4.71) |

**Table SM3. 2. Pairwise comparisons with Bonferroni adjustment (Switzerland, 2026).**

**Information and awareness-raising**

**contrast	estimate	SE df t.ratio p.value**

cluster 1 - cluster 2	-0.135 0.141 106 -0.957 1.0000

cluster 1 - cluster 3	-1.492 0.154 106 -9.675 <.0001

cluster 1 - cluster 4	-1.849 0.163 106 -11.372 <.0001

cluster 2 - cluster 3	-1.357 0.167 106 -8.118 <.0001

cluster 2 - cluster 4	-1.714 0.175 106 -9.798 <.0001

cluster 3 - cluster 4	-0.357 0.186 106 -1.920 0.3454

**Interventions in the built and natural environment**

**contrast	estimate	SE df t.ratio p.value**

cluster 1 - cluster 2	-0.698 0.252 106 -2.769 0.0398

cluster 1 - cluster 3	-1.221 0.276 106 -4.423 0.0001

cluster 1 - cluster 4	-1.809 0.291 106 -6.211 <.0001

cluster 2 - cluster 3	-0.524 0.299 106 -1.749 0.4991

cluster 2 - cluster 4	-1.111 0.313 106 -3.545 0.0035

cluster 3 - cluster 4	-0.587 0.333 106 -1.762 0.4853

**Interventions promoting healthy lifestyles and strengthening social cohesion**

**contrast	estimate	SE df t.ratio p.value**

cluster 1 - cluster 2	-1.80 0.639 106 -2.814 0.0350

cluster 1 - cluster 3	-3.31 0.701 106 -4.724 <.0001

cluster 1 - cluster 4	-5.53 0.739 106 -7.488 <.0001

cluster 2 - cluster 3	-1.51 0.760 106 -1.990 0.2950

cluster 2 - cluster 4	-3.73 0.795 106 -4.696 <.0001

cluster 3 - cluster 4	-2.22 0.845 106 -2.629 0.0591
